# Supplementary material for: Laser Er:YAG-Assisted Debonding May Be a Viable Alternative to the Conventional Method for Monocrystalline Ceramic Brackets
Source: Int J Environ Res Public Health. 2022 Nov 6;19(21):14564. doi: 10.3390/ijerph192114564 (PMC9657488; doi:10.3390/ijerph192114564)
Supplement: Supplementary file 1 [file ijerph-19-14564-s001.zip › ijerph-1985607-supplementary.pdf]

SUPPLEMENTARY MATERIALS

Table S1. The pain scores at Wong-Baker FACES Pain Rating Scale, immediately after debonding and the needed time (sec) for removing the brackets from the surface of the tooth.

Type of debonding	Tooth n=30	Pain Scores	Time (sec.)	Type of debonding	Tooth n=30	Pain Scores	Time (sec.)
Er:YAG LASER DEBONDING	1.1	2	1	CONVENTIONAL DEBONDING	2.1	2	2
	1.2	0	1,2		2.2	4	2
	1.3	0	0,5		2.3	2	2,4
	1.1	0	0,9		2.1	2	1,6
	1.2	0	0,5		2.2	6	1,5
	1.3	0	0,9		2.3	2	2
	1.1	2	1,2		2.1	4	1,2
	1.2	0	1		2.2	4	1,5
	1.3	0	1,1		2.3	2	1
	1.1	0	0,9		2.1	4	0,5
	1.2	2	1		2.2	2	1
	1.3	0	1,1		2.3	2	1,3
	1.1	0	1,2		2.1	6	2
	1.2	0	0,8		2.2	2	2,3
	1.3	0	1		2.3	4	3
	1.1	2	0,8		2.1	2	1,6
	1.2	0	1		2.2	4	0,9
	1.3	0	1,5		2.3	2	1,2
	1.1	2	1		2.1	2	1,3
	1.2	0	0,7		2.2	6	1
	1.3	0	1		2.3	2	2
	1.1	0	0,5		2.1	4	2,5
	1.2	0	0,7		2.2	2	2,2
	1.3	0	0,8		2.3	2	1,8
	1.1	2	1,2		2.1	2	1,4
	1.2	0	1		2.2	4	0,9
	1.3	0	1,4		2.3	2	1,3
	1.1	2	1		2.1	2	1,6
	1.2	0	0,8		2.2	6	2
	1.3	0	0,5		2.3	2	2,1

Table S2 Pulp blood flow mean values (M) and standard deviation (**SD)** recorded at the three moments of the study using laser Doppler flowmetry.

Type of T	Tooth n=30	Before debonding		Imediatelly after debonding		7 days after debonding		Type of T	Tooth n=30	Before debonding		Imediatelly after debonding		7 days after debonding	
		M (PU)	SD	M (PU)	SD	M (PU)	SD			M (PU)	SD	M (PU)	SD	M (PU)	SD
Er:YAG LASER DEBONDING	1.1	10.6	1.6	6.4	1.2	14.5	2.2	CONVENTIONAL DEBONDING	2.1	8.5	1.1	7.2	1.4	10.0	1.9
	1.2	15.1	2.0	18.3	2.9	22.5	2.8		2.2	9.4	1.3	12.1	1.8	17.7	2.6
	1.3	12.4	1.7	15.6	2.3	29.7	3.5		2.3	14.6	1.7	11.5	1.6	25.1	3.8
	1.1	9.9	1.4	6.7	1.1	12.0	2.0		2.1	12.6	1.9	8.3	1.4	14.7	3.5
	1.2	12.1	2.0	11.1	1.7	16.7	3.8		2.2	17.2	2.3	23.5	3.9	25.0	5.4
	1.3	11.9	1.9	15.3	2.8	18.3	3.8		2.3	9.2	1.4	16.5	3.1	17.0	3.3
	1.1	8.4	1.5	8.4	1.5	10.3	1.8		2.1	15.3	2.1	14.3	3.7	13.8	1.9
	1.2	17.4	2.6	16.1	2.3	14.6	2.3		2.2	15.8	2.5	22.3	5.9	11.3	1.7
	1.3	17.0	2.7	9.5	2.1	13.5	2.2		2.3	16.6	2.8	13.6	2.2	13.2	2.5
	1.1	8.1	1.3	9.7	3	10.3	2.6		2.1	9.1	2	9.3	2.5	10.6	1.8
	1.2	9.3	1.4	9.3	1.5	11.3	2.5		2.2	8.6	3	9.9	3.2	10.3	2
	1.3	8.4	1.7	9.9	2.2	10.3	1.9		2.3	13	2.7	12.4	3.6	17.6	2.8
	1.1	11.6	3.9	10.4	3.2	11.4	2.6		2.1	11.4	2.6	12.6	2.2	13.2	2.2
	1.2	10.3	2	12.6	3.9	15.2	3.4		2.2	13	3.6	8.6	3	11.4	2.6
	1.3	11.3	2.5	14.6	3.1	16.3	3.7		2.3	13.1	2.5	14.5	2.8	15.2	2.4
	1.1	9.4	1.3	12.1	1.8	17.7	2.6		2.1	11.6	1.6	7.4	1.2	14.7	3.5
	1.2	12.4	1.7	15.6	2.3	29.7	3.5		2.2	9.5	1.1	7.2	1.4	10.0	1.9
	1.3	9.9	1.4	8.7	1.2	12.0	2.0		2.3	9.4	1.3	12.1	1.8	17.7	2.6
	1.1	12.1	2.0	11.1	1.7	16.7	3.8		2.1	14.6	1.7	11.5	1.6	21.1	3.8
	1.2	11.9	1.9	15.3	2.8	18.3	3.8		2.2	11.6	1.9	8.3	1.5	14.7	3.5
	1.3	10.6	1.6	12.4	1.3	14.5	2.2		2.3	13.2	2.3	13.5	3.0	25.0	5.4
	1.1	15.1	2.0	18.3	2.9	22.5	2.8		2.1	9.2	1.4	10.5	3.1	15.0	3.2
	1.2	8.4	1.7	10.9	2.3	15.3	1.9		2.2	15.3	2.1	14.3	3.7	13.8	1.9
	1.3	11.6	3.9	10.4	3.2	11.4	2.6		2.3	12.8	2.5	12.3	5.9	13.3	1.8
	1.1	10.3	2	12.6	3.9	15.2	3.4		2.1	16.6	2.8	13.6	2.2	13.2	2.5
	1.2	8.1	1.3	9.7	3	10.3	2.6		2.2	9.1	2.1	9.3	2.5	10.6	1.8
	1.3	9.3	1.4	9.3	1.5	11.3	2.5		2.3	8.6	2.6	9.9	3.2	10.3	2
	1.1	11.3	2.5	14.6	3.1	16.3	3.7		2.1	15	2.7	12.2	3.6	16.6	2.7
	1.2	17.2	2.3	23.5	3.9	25.0	5.4		2.2	11.6	2.6	15.6	2.2	13.2	2.2
	1.3	13.2	2.3	18.5	3.0	25.0	5.4		2.3	12.4	3.2	8.6	2.1	11.4	2.5

Abbreviations: T - treatment

PU –perfusion units
